# Prognostic Value and Stage/Nodal Correlates of Serum and Salivary CRP, CYFRA 21-1, and SCC-Ag Biomarkers in Oral Squamous Cell Carcinoma: A Systematic Review and Meta-Analysis

**DOI:** 10.3390/medsci14060610

**Published:** 2026-09-27

**Authors:** Balen Hamid Qadir, Mohammed Khalid Mahmood, Hazha Abdulla Mohammed Ameen, Yad Mariwan Mohammed Amin, Ariwan Othman Saeed, Florence Carrouel, Romain Lan

**Affiliations:** 1 Department of Dentistry, Komar University of Science and Technology, Sulaimani 46001, Iraq; 2 Bio-Cultural Anthropology, Law, Ethics and Health Laboratory (ADES), Faculty of Medical and Paramedical Sciences, Aix-Marseille University, French National Center of Scientific Research (CNRS), French Blood Establishment (EFS), 13005 Marseille, France; 3 College of Medicine, Sulaymaniyah University, Sulaymaniyah 46001, Iraq; 4 Health Systemic Process (P2S) UR4129, Université Lyon 1, 69008 Lyon, France; 5 Department of Oral Surgery, Timone Hospital, Assistance Publique—Hôpitaux de Marseille, 13385 Marseille, France

**Keywords:** oral cancer, oral squamous cell cancer, biomarkers, CYFRA 21-1, SCC-Ag, CRP

## Abstract

**Background:** Oral squamous cell carcinoma (OSCC) has a relatively poor survival, and TNM staging alone offers limited prognostic discrimination. C-reactive protein (CRP) is a non-specific inflammatory marker and is one of the most studied candidate prognostic biomarkers of OSCC, while CYFRA 21-1 and squamous cell carcinoma antigen (SCC-Ag) are tumor-derived keratin proteins reflecting malignant cell turnover, often examined alongside CRP. However, no prior review has synthesized the value of these three markers together in OSCC. **Methods:** Following the PRISMA 2020 guidelines, PubMed, Embase, and Web of Science were searched for studies reporting an extractable hazard ratio (HR), odds ratio, or comparable association of serum and/or salivary CYFRA 21-1, SCC-Ag, and/or CRP with disease-free survival (DFS), recurrence, or overall survival (OS) in OSCC. HRs were pooled using Knapp–Hartung random-effects models, wherever at least two independent cohorts contributed; salivary CYFRA 21-1 and recurrence were pooled as a standardized mean difference (SMD). Studies reporting only cross-sectional associations with tumor stage, grade, or metastasis and non-independent (same-registry) studies were synthesized descriptively following Synthesis without Meta-analysis (SWiM) guidance. Risk of bias was assessed with QUIPS and certainty of evidence with the GRADE framework. **Results:** Twenty-eight studies were included (21 quantitative, 7 narrative), representing ~3500 study participants across published cohorts, with some overlap between studies. For OS, two independently pooled single-marker estimates were both non-significant: serum SCC-Ag alone (n = 2, HR 1.43, 95% CI: 0.09–23.14, *p* = 0.35, I^2^ = 66%) and serum CRP alone (n = 5, HR 1.33, 95% CI: 0.89–1.99, I^2^ = 52%). A further seven non-independent, same-registry studies reporting combined SCC-Ag + CRP or single-marker models ranged from HR 1.82 to 11.11, directionally consistent with the pooled estimates. For DFS, pooled serum CYFRA 21-1 alone gave HR 1.61 (95% CI: 0.39–6.61; *p* = 0.15, I^2^ = 0%), with six further non-independent or single-study estimates (HR 1.38–4.05) in the same direction. For recurrence, pooled salivary CYFRA 21-1 gave an SMD of Hedges’ g = 0.79 (95% CI: 0.04–1.55; *p* = 0.048, I^2^ = 0%). All outcomes were rated very low certainty by GRADE. **Conclusions:** Within the limitations of the study and the small number of contributing studies, salivary CYFRA 21-1 showed some prognostic value for recurrence, while pooled estimates for serum CRP alone, serum SCC-Ag alone, and serum CYFRA 21-1 alone for DFS all trended toward increased risk but did not reach statistical significance. Given the very low evidence certainty, these findings are hypothesis-generating and require further validation in independent, multicenter, prospective cohorts before clinical application.

## 1. Introduction

Oral squamous cell carcinoma (OSCC) remains one of the most fatal head and neck malignancies, with recurrence rates of up to 30–40% after curative treatment and five-year survival rates that have not improved substantially over recent decades [1,2]. Conventional TNM staging is based on anatomical tumor extent and offers limited discriminatory power for individual recurrence risk [3]. This motivated growing interest in circulating and salivary biomarkers that might refine prognostication beyond stage alone.

C-reactive protein (CRP), which is a non-specific acute-phase reactant, has also been examined as a candidate prognostic marker in OSCC [4]. In addition, Cytokeratin 19 Fragment Antigen 21-1 (CYFRA 21-1), a soluble fragment of cytokeratin 19, and squamous cell carcinoma antigen (SCC-Ag), often examined alongside CRP, are the most extensively studied candidate tumor-derived markers, with individual cohort studies since the mid-1990s reporting associations between elevated concentrations and tumor recurrence or reduced survival [5,6].

Several studies have examined the relation between these biomarkers and OSCC prognosis. A recent meta-analysis pooled 6 CRP studies in oral cancer and reported a significant adverse association for both OS (HR 1.80, 95% CI 1.11–2.92) and DFS (HR 1.81, 95% CI 1.28–2.56); however, they pooled at least two same-registry studies as independent records [7]. Another review broadly surveyed salivary proteomic, transcriptomic, and metabolomic biomarkers for OSCC prognosis, but was explicitly narrative and qualitative in design, was restricted to salivary specimens, and included only one CYFRA 21-1 study [8]. However, the largest existing head-and-neck-specific meta-analysis of 1901 cases found SCC-Ag correlated with TNM stage but could not establish it as a predictive marker for OS or DFS in HNSCC [9]. In contrast, a cross-sectional study directly comparing paired serum and salivary tumor markers in 21 OSCC patients found CYFRA 21-1 among the markers most substantially elevated in saliva [10].

CYFRA 21-1 and SCC-Ag are both tumor-derived keratin proteins reflecting malignant cell turnover [11], whereas CRP is a non-specific host inflammatory marker [12]. These three biomarkers are combined in this review not because they share a biological mechanism, but because primary studies consistently report them together as a combined tumor-burden and systemic-inflammation panel and, in our own included evidence, six of the eleven quantitative cohort studies reported two or more of these markers jointly [13]. To our knowledge, no prior review has specifically and quantitatively pooled CYFRA 21-1, SCC-Ag, and CRP across both serum and saliva in OSCC. We therefore conducted a systematic review and meta-analysis to synthesize the prognostic value of these biomarkers, individually and in combination, in the recurrence and survival of OSCC.

## 2. Methods

### 2.1. Protocol

This review was conducted according to the PRISMA 2020 statement [14]. The protocol was registered beforehand in the PROSPERO database under the registration number CRD420261473595. The narrative part of the synthesis was reported in accordance with the SWiM guideline [15].

### 2.2. Eligibility Criteria

This review asked whether serum and/or salivary CRP, CYFRA 21-1, and SCC-Ag, alone or combined, can predict OSCC prognosis. Eligible studies enrolled adults with histopathologically confirmed OSCC (population), measured serum and/or salivary CYFRA 21-1, SCC-Ag, and/or CRP status (exposure), compared elevated versus normal or continuous marker status (comparator), and reported recurrence, DFS, or OS (outcome), assessed at or near diagnosis with unrestricted follow-up (time).

Studies were included if they used a cohort, cross-sectional, or trial design; enrolled histopathologically confirmed oral cavity OSCC patients; assessed CYFRA 21-1, SCC-Ag, and/or CRP prognostically; and reported an extractable effect size for recurrence or survival, or, failing that, a qualitative association with stage, grade, or metastasis. Although cross-sectional associations with stage, grade, or metastasis are not direct prognostic outcomes, they were retained only as secondary, descriptive clinicopathological evidence and are reported separately from the prognostic (recurrence/survival) analyses. No restrictions were placed on setting, language, or publication date.

Studies were excluded if the population mixed oral cavity with other head/neck or extra-oral sites without separable data; if the outcome was diagnostic accuracy only; if the marker’s association with the outcome could not be isolated as its own extractable effect size; if the study was a case report, abstract, review, or preclinical study; or if no effect size or relevant qualitative prognostic/clinicopathological association could be extracted. Studies sharing an overlapping patient registry were retained as non-independent, descriptive evidence rather than excluded or formally pooled.

### 2.3. Search Strategy

PubMed, Embase, and Web of Science were searched from inception to August 2026, combining two concept blocks: (i) oral squamous cell carcinoma and (ii) the CRP, CYFRA 21-1 and SCC-Ag biomarkers. Reviews, editorials, letters, case reports, and conference abstracts were excluded at the database level using publication type filters, as these publication types do not report an extractable effect size or individual patient data. Reference lists of included studies were searched for additional eligible studies. The full search strategy is presented in Supplementary Table S1.

### 2.4. Study Selection

The Zotero (9.0.6, 64-bit) software was used to import all retrieved entries and remove duplicates before title and abstract screening. Two independent reviewers, M. M. and Y. A., selected studies in two rounds. Using eligibility criteria, Part 1 of the two-part study selection approach screened abstracts and titles for suitable studies. Part 2 involved a thorough full-text article eligibility screening of possibly qualifying research. Studies meeting all qualifying criteria were included. The two reviewers resolved screening disputes. When agreement could not be reached, R. L., a third reviewer, resolved the disagreement.

### 2.5. Data Extraction

Two reviewers extracted data independently using a standardized form, including study characteristics (author, year, country, design), population size, specimen type (serum/salivary), marker(s) assessed, cut-off values, and prognostic outcomes (recurrence, DFS, OS), along with reported effect sizes and 95% confidence intervals. Where survival or recurrence data were unavailable, associations with tumor stage, grade, or metastasis were extracted as secondary clinicopathological evidence and were included only in the narrative synthesis. Studies sharing an overlapping patient registry were treated as non-independent. Discrepancies were resolved by discussion between reviewers.

### 2.6. Quality Assessment

Two reviewers assessed risk of bias independently using the Quality in Prognosis Studies (QUIPS) methodology, which examines study participation, attrition, prognostic factor measurement, outcome measurement, confounding, and statistical analysis and reporting. Each domain was graded as low, moderate, or high risk of bias, per QUIPS guidelines for domain-specific judgements. Although this tool does not suggest an overall score, for the sake of subgroup comparison, we applied the following rule to estimate the total score of a paper: overall risk was rated high if any domain was high, moderate if the highest domain was moderate, otherwise low [16]. Disagreements were resolved by discussion.

### 2.7. Evidence Synthesis

Where at least two independent cohorts reported a comparable marker–outcome combination, HR were pooled on the log scale using Knapp–Hartung random-effects model, with heterogeneity quantified by I^2^; salivary CYFRA 21-1 and recurrence were pooled as a standardized mean difference (Hedges’ g). Studies sharing an overlapping patient registry, or contributing only a single estimate, could not be pooled and were instead synthesized narratively as directional, non-independent evidence, compared descriptively rather than statistically combined. Certainty of evidence was assessed using GRADE for the pooled effect sizes [17], while the certainty for the non-pooled component was assessed using the modified GRADE for rating the certainty in evidence in the absence of a single estimate of effect [18].

## 3. Results

### 3.1. Selection of Studies

The database query produced 1090 entries. Following the elimination of 302 duplicates, 788 entries were evaluated based on their titles and abstracts. Out of them, 745 were deemed irrelevant, resulting in 43 full-text papers evaluated for eligibility. In the comprehensive evaluation, 15 records were omitted (see Supplementary Table S2 for reasons), leading to 28 studies used in the ultimate analysis [4,13,19,20,21,22,23,24,25,26,27,28,29,30,31,32,33,34,35,36,37,38,39,40,41,42,43,44], consisting of 21 for quantitative and 7 for narrative synthesis. Figure 1 illustrates the procedure for selecting studies.

### 3.2. Characteristics of the Included Studies

The included studies were published between 1990 and 2026 and originated from eleven countries: Taiwan (n = 10, all sharing the Chang Gung Memorial Hospital registry with overlapping enrolment windows), Japan (n = 4), India (n = 4), China (n = 2), Germany (n = 2), and North Macedonia, Poland, Switzerland, Austria, Korea, and the United Kingdom (n = 1 each). Most were retrospective cohorts (n = 20); four were prospective cohorts and four were cross-sectional. Individual study sample sizes ranged from 20 to 343 patients, while the total number of participants across the included studies was approximately 3496, with some overlap between publications sharing the same registry. Serum was the specimen assessed in 24 studies, while four evaluated salivary (or paired salivary and serum) concentrations. CRP was the most frequently studied marker (n = 20 studies), most often assessed alone (n = 15); SCC-Ag was assessed in nine studies, either alone or combined with CRP and/or CYFRA 21-1; CYFRA 21-1 was assessed in five studies, alone or combined with SCC-Ag and/or CRP. Reported outcomes included OS, DFS, and recurrence in twenty-one studies, and cross-sectional associations with tumor stage, histological grade, or metastasis in seven studies. Adjustment varied considerably across studies; some multivariable models controlled for 4–6 factors (stage, differentiation, tumor depth, nodal status, comorbidity), a couple of studies deliberately separated the marker from composite indices to avoid multicollinearity, some reported a significant adjusted result without ever listing its covariates, and one used no adjustment at all (univariate only). This diversity in the adjustment is itself a source of heterogeneity worth naming. Supplementary Table S4 presents the full detail about the adjustment model and status of each included study. Table 1 contains the key characteristics of the included studies.

### 3.3. Quality Assessment Results

Of the 28 included studies, three were judged as having overall low risk of bias, thirteen as having moderate risk, and twelve as having high risk of bias. Prognostic factor measurement was the best-performing domain, rated low risk in the great majority of studies, reflecting standardized immunoassay methods. Confounding was the most frequently problematic domain overall and only seven studies adequately accounted for confounders such as age, stage, or treatment. Study attrition was the second most consistently weak domain, most often rated moderate or unable to be assessed, since follow-up completeness was rarely reported. Outcome measurement and statistical analysis and reporting were generally low risk among the quantitative cohort studies but moderate among the cross-sectional and narrative studies. Figure 2 shows the details of the quality assessment for each study.

### 3.4. Evidence Synthesis Results

The pooled and non-pooled single-study estimates are presented in Figure 3 and Figure 4.

#### 3.4.1. Overall Survival

Five independent cohorts [36,37,38,41,44] assessed serum CRP alone as a prognostic marker for OS and pooled to an HR of 1.33 (95% CI: 0.89–1.99; I^2^ = 52%), not reaching statistical significance.

Two independent cohorts [19,20] reported serum SCC-Ag alone as a prognostic marker for OS, contributing to a pooled HR of 1.43 (95% CI: 0.09–23.14; I^2^ = 66%), again not reaching statistical significance.

Seven further studies reporting combined SCC-Ag + CRP or CRP-alone models could not be pooled because they shared an overlapping registry. All but one of these registry studies reported a significant HR ranging from 2.24 to 11.11 [4,24,25,27,33,43]. The single exception, a CRP-alone model from the same registry, reported a non-significant association (HR 1.82, 95% CI: 0.50–6.61) [26]. Despite this variability in magnitude, the pooled and non-pooled effect sizes from this registry consistently favored an adverse prognostic association.

#### 3.4.2. Disease-Free Survival

Two independent cohorts reported serum CYFRA 21-1 alone and contributed to a pooled HR of 1.61 (95% CI: 0.39–6.61; I^2^ = 0%), not reaching statistical significance.

Six further non-independent or single-study models reported combined or CRP-alone/three-marker models. Among these, four were significant [4,24,26,43], whereas a three-marker model reported a non-significant association (HR 2.41, 95% CI: 0.94–6.22) [21] and a CRP-alone model in which CRP was a secondary co-variable (HR 1.38, 95% CI: 0.57–3.35) [41]. The pooled DFS estimate, like the OS estimates, did not reach significance; the non-pooled registry findings, however, were directionally consistent with an adverse prognostic association.

#### 3.4.3. Recurrence

Salivary CYFRA 21-1 was significantly higher in patients who subsequently recurred than in those who did not, pooled across two independent cohorts as an SMD of Hedges’ g = 0.79 (95% CI: 0.04–1.55; *p* = 0.048, I^2^ = 0%). It should be noted that this pooled SMD represents the magnitude of difference in marker concentration between patients who did and did not later recur, rather than a time-to-event hazard, and is therefore not directly comparable in magnitude to the HR.

A third, independent prospective cohort could not be pooled with this estimate, since it used a different effect measure, but reported that elevated baseline salivary CYFRA 21-1 (adjusted HR 4.10, 95% CI: 1.59–10.54) and a large post-treatment reduction (adjusted HR 3.00, 95% CI: 1.21–7.44) both independently predicted recurrence-free survival, while TNM stage and nodal status were not significant in the same model [28]. Despite the differing effect measures, both bodies of evidence pointed the same way: higher baseline salivary CYFRA 21-1 was consistently associated with worse recurrence outcomes.

A separate single-cohort analysis examined serum CRP in relation to recurrence and metastasis. Elevated CRP was associated with a higher recurrence rate (RR 1.77, 95% CI: 1.06–2.94) but was not associated with metastasis (RR 0.55, 95% CI: 0.28–1.09) [32]. It is important to note that this was a crude proportion that did not account for differences in follow-up time.

#### 3.4.4. Stage and Grade Correlates

One study reported 42.6% SCC-Ag positivity correlating with T-stage, in a population confirmed to be oral-cavity only; notably, recurrence was numerically lower in the elevated-marker group, an inverse pattern not seen in the quantitative cohorts [30].

Five further studies assessed serum CRP. Serum CRP correlated with advanced tumor stage in a cross-sectional comparison [31], and, in a separate case–control study against 30 healthy controls, correlated significantly with positive nodal status [34]. Another study reported CRP rising stepwise across a three-group comparison of healthy controls, oral premalignant lesions, and OSCC, and additionally found CRP increasing significantly with clinical stage within the OSCC group itself [40]. Two remaining studies each found CRP significantly correlated with local tumor burden [35,42] but neither found CRP associated with OS in the same cohort, distinguishing correlation with tumor burden from independent prognostic value.

#### 3.4.5. Descriptive Stratified Analyses of Non-Pooled Evidence

To assess whether the adverse prognostic association was consistent across the non-pooled studies rather than driven by a specific subset, all studies reporting a hazard ratio for OS or DFS that could not be pooled (n = 13: 7 for OS, 6 for DFS) were compared descriptively by sample size, study quality, marker combination, and enrolment era. The association remained directionally consistent across every stratification: all 13 estimates were directionally consistent with an adverse association, with no stratum reversing direction (Supplementary Figures S1–S4).

#### 3.4.6. Evidence Certainty

For each outcome, GRADE certainty was assessed for the combined body of evidence: pooled estimate(s) together with non-independent or standalone evidence for that outcome. The OS, DFS, and recurrence outcomes were each rated very low certainty, reflecting risk of bias from the overlapping registry and, for OS, from most pooled CRP studies treating CRP as a secondary rather than primary exposure; indirectness between single-marker and composite-marker exposures; and imprecision (Supplementary Table S3).

## 4. Discussion

This systematic review synthesizes the prognostic value of serum/salivary CRP, CYFRA 21-1, and SCC-Ag in OSCC. Salivary CYFRA 21-1 emerged as the only borderline statistically significant signal associated with OSCC recurrence. Serum CYFRA 21-1 alone for DFS, serum SCC-Ag alone, and serum CRP for OS did not reach statistical significance though point estimates favored an adverse association, with substantial heterogeneity. Moreover, the non-pooled composite SCC-Ag + CRP models and the CRP-alone models were directionally concordant, though their HR magnitudes are not directly comparable given differences in model composition and, in several cases, overlapping cohorts. This may indicate that tumor-derived and host-response markers capture complementary prognostic information. Alternatively, it may simply reflect two independent opportunities to cross a significance threshold, a distinction this review cannot resolve.

The findings of this review sit within a modest body of directly comparable literature. A previous review pooled 6 CRP studies in OSCC and reported a significant adverse association for both OS (HR 1.80, 95% CI 1.11–2.92) and DFS (HR 1.81, 95% CI 1.28–2.56) [7]. This is a larger and significant effect compared to the present review’s independent CRP-alone OS pool (HR 1.33, 95% CI 0.89–1.99). This discrepancy is likely explained by the previous review pooling at least two same-registry studies as independent, whereas the present review excluded them from pooling. However, both reviews agree in direction, with elevated CRP consistently associated with worse prognosis. CRP’s adverse prognostic association is broadly replicated across solid tumors: a systematic review of 271 studies found elevated CRP predictive of prognosis in 90% of reports, most consistently in gastrointestinal and renal malignancies [45], making its association with OSCC outcomes in this review consistent with the wider oncology literature despite its lack of tumor specificity.

Pellegrini et al. [8] surveyed the broader salivary biomarker landscape in OSCC but treated CYFRA 21-1 as only one candidate among many other omics markers, including only one CYFRA 21-1 study [22] in their narrative review of diagnosis, grade, and recurrence correlations. A dedicated OSCC-specific meta-analysis of CYFRA 21-1 addressed diagnostic accuracy only (pooled sensitivity 0.71, specificity 0.88, AUC 0.927), not prognosis [46]. The most directly comparable prior synthesis is a broader head-and-neck cancer meta-analysis, which pooled seven prognostic studies across all head-and-neck subsites and reported CYFRA 21-1 associated with shorter OS (HR 1.33, 95% CI: 1.13–1.56) and DFS (HR 1.48, 95% CI: 1.10–1.97) [47]. The pooled CYFRA 21-1 estimate for DFS in our oral-cavity-specific analysis (HR 1.61 95% CI 0.39–6.61) was of similar magnitude to the DFS estimate reported in this broader head-and-neck meta-analysis (HR 1.48), though our estimate, which was based on only two contributing cohorts, was not statistically significant [47]. In other cancers, CYFRA 21-1’s prognostic value is well established in non-small-cell lung cancer, where meta-analyses of 2063 [48] and 6394 patients [49] both confirmed elevated pretreatment CYFRA 21-1 as an adverse prognostic marker (pooled HR ranging from 1.60 to 1.88 across stage and treatment subgroups).

Our null finding of SCC-Ag for OS is consistent with the largest existing head-and-neck-specific synthesis of SCC-Ag—an 11-study meta-analysis (1901 cases) that similarly found SCC-Ag correlated with TNM stage but could not establish it as a predictive marker for OS or DFS in HNSCC [9]. However, a 61-study meta-analysis in cervical squamous cell carcinoma found elevated pretreatment SCC-Ag associated with recurrence (pooled HR 2.23, 95% CI: 2.03–2.45) [50] likely reflecting the much larger evidence base available in cervical cancer.

CYFRA 21-1 and SCC-Ag are structural epithelial proteins released during malignant keratinocyte turnover, apoptosis, and necrosis, directly reflecting tumor burden [51], while CRP, a non-specific acute-phase reactant, reflects the systemic inflammatory response to the tumor [6]. Salivary CYFRA 21-1’s significant association with recurrence is consistent with its direct contact with the oral cancer lesion, shown empirically to elevate salivary CYFRA 21-1 concentrations well above serum levels in OSCC patients [10].

A statistically significant HR demonstrates an association between a biomarker and outcome, not clinical prognostic utility. It does not show that the biomarker improves prediction beyond TNM stage or other established clinical variables. None of the included studies reported discrimination, calibration, or reclassification metrics, and they have not validated multivariable prognostic models incorporating these markers alongside standard clinical predictors. Such analyses would be required before any claim of clinical usefulness could be made. Thus, the findings of this review should be interpreted as evidence of association only.

Most quantitative evidence originated from a single, overlapping Taiwanese registry, limiting genuinely independent effect sizes to a few per subgroup and limiting the statistical power. This small number of independent studies also makes heterogeneity estimates unstable, and such values should not be read as evidence of true homogeneity. Marker cut-offs, assay platforms, and covariate adjustment sets varied across studies, limiting direct comparability of effect estimates. Eligibility criteria also admitted cross-sectional stage/grade/metastasis associations alongside genuinely prognostic outcomes; these are reported separately but draw on overlapping literature. Finally, publication bias could not be formally assessed given the small number of studies per outcome.

## 5. Conclusions

In conclusion, this systematic review found a predominantly adverse association across the outcomes examined for all three biomarkers, including a directional signal for salivary CYFRA 21-1 and recurrence based on only two independent cohorts. Although all three biomarkers trended toward increased risk, none reached statistical significance in their independently pooled estimates, despite CRP being the most frequently studied marker in the included evidence. Given the small number of independent studies, the substantial heterogeneity, and the very low certainty of the evidence across all outcomes, these findings should be regarded as candidate associations requiring independent validation, rather than as evidence of comparative prognostic performance among CRP, SCC-Ag, and CYFRA 21-1. Further research in adequately powered, independent cohorts is needed before recommending the use of any of these markers in the prognosis of OSCC.

## Figures and Tables

**Figure 1:**
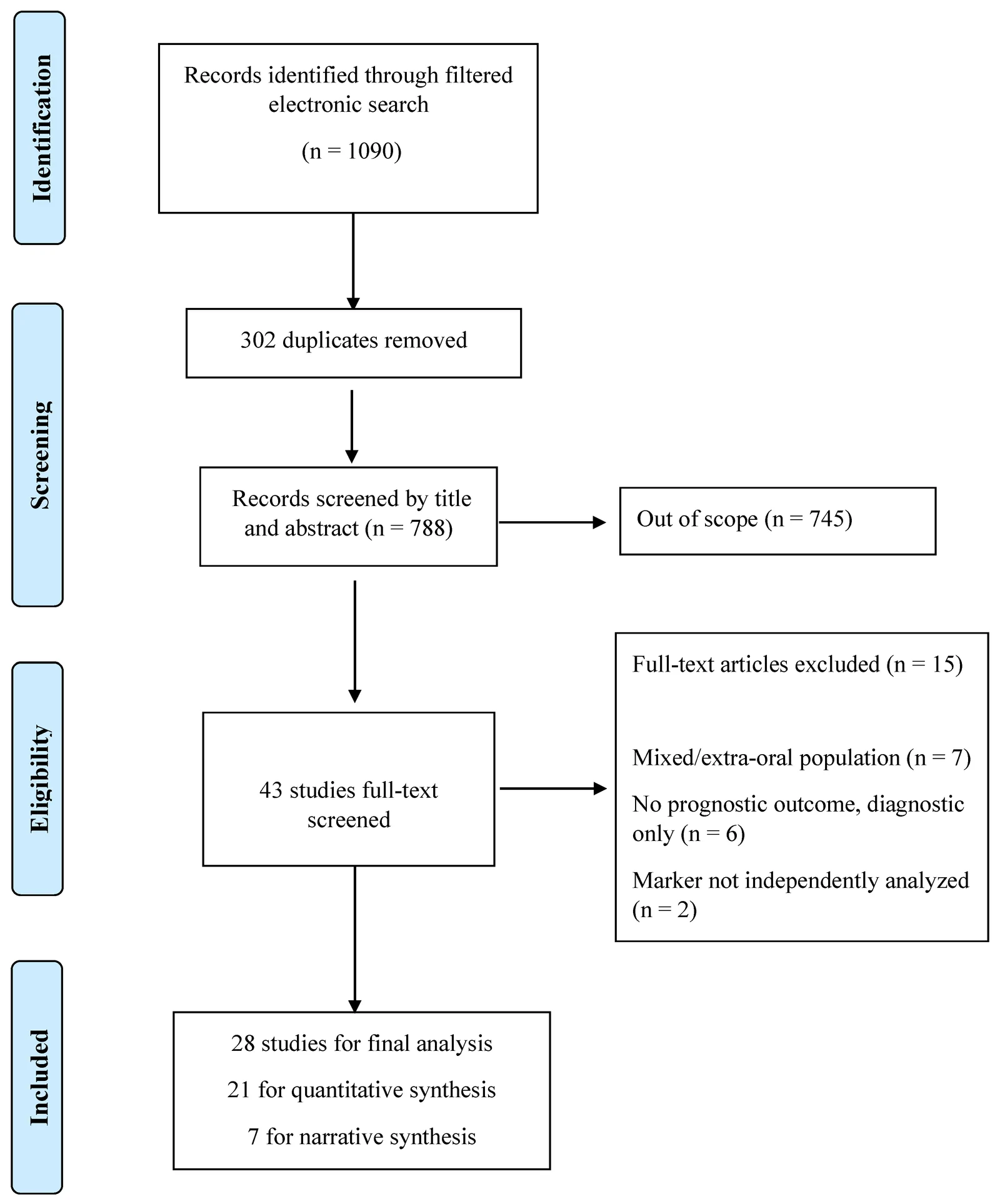
Study selection process.

**Figure 2:**
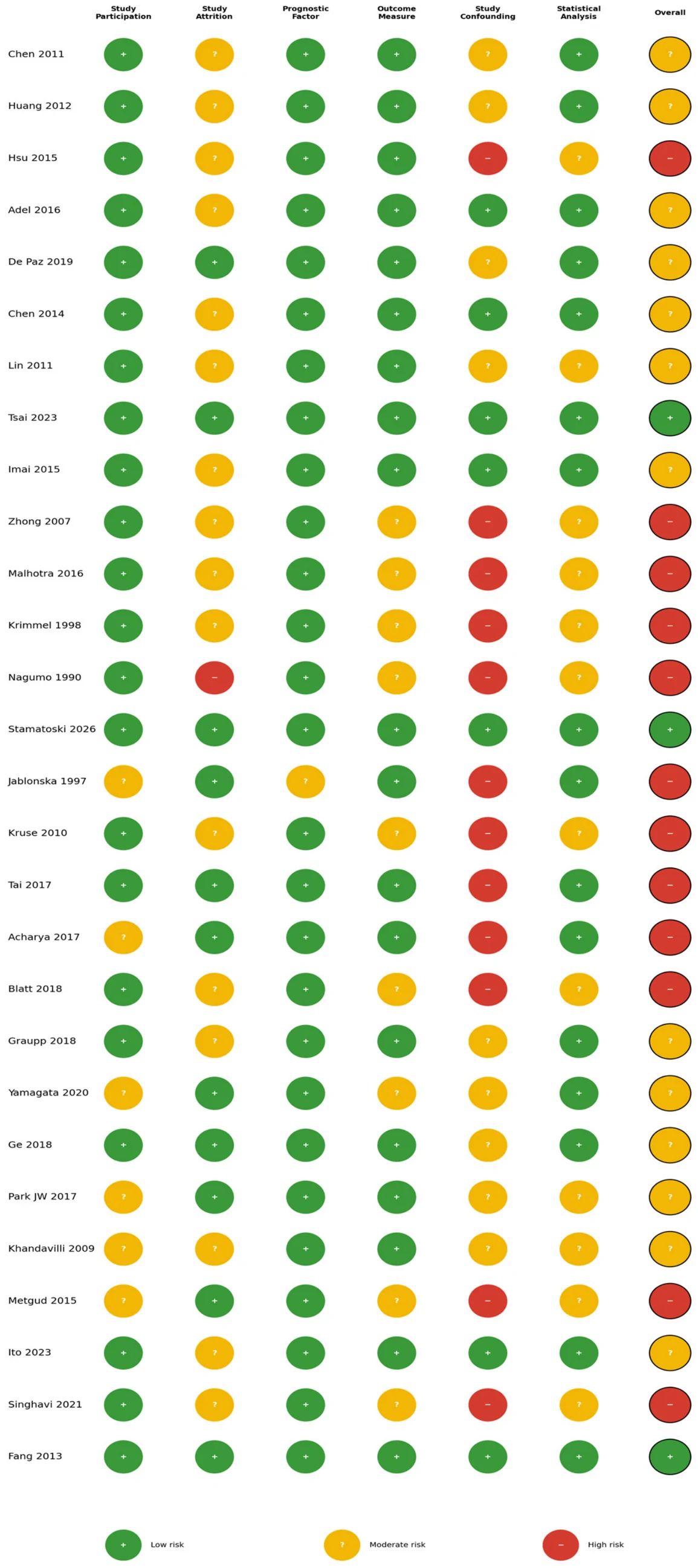
Risk of bias by QUIPS domains [16].

**Figure 3:**
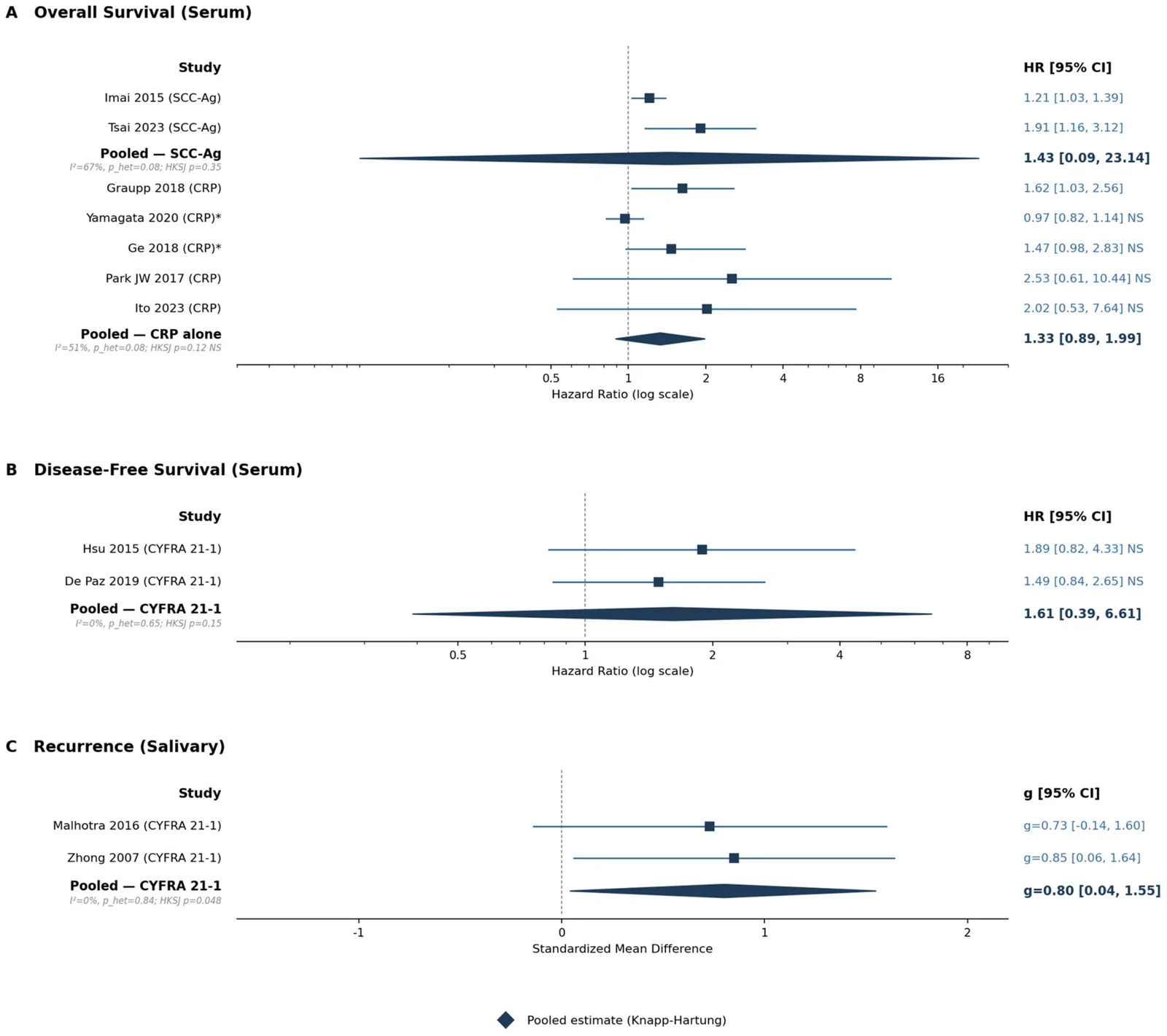
Prognostic value of serum/salivary CRP, SCC-Ag, and CYFRA 21-1 in oral squamous cell carcinoma (pooled data). * These data represent the multivariate analysis; the univariate findings of these studies were significant.

**Figure 4:**
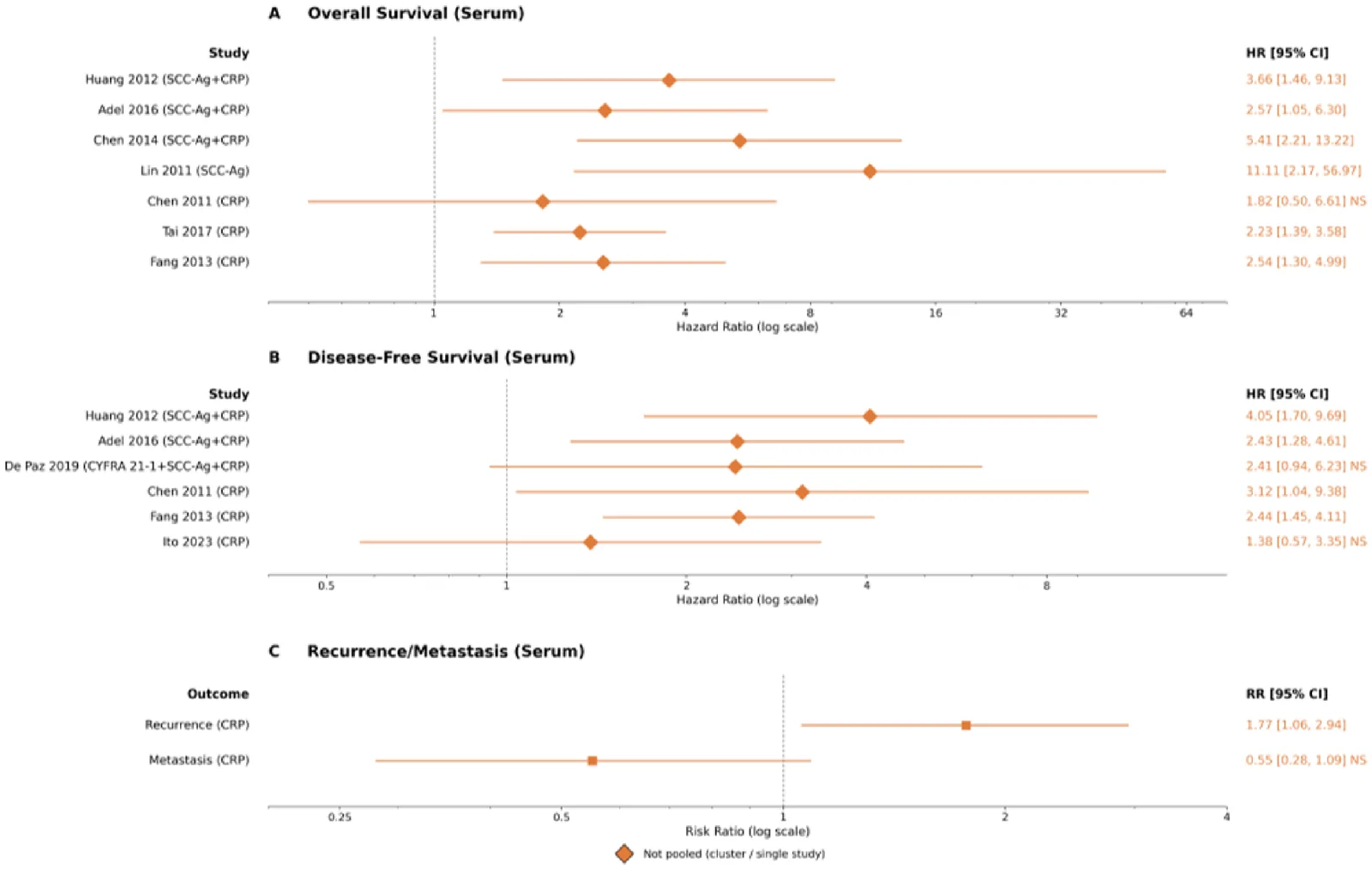
Prognostic value of serum/salivary CRP, SCC-Ag, and CYFRA 21-1 in oral squamous cell carcinoma (non-pooled, single study data).

**Table 1:** Characteristics of the included studies.

Study	Year	Country	Evidence-Type	N	Design	Specimen	Marker(s)	Outcome(s)	Key Findings
Imai [19]	2015	Japan	Prognostic (outcome-associated)	78	Retrospective cohort	Serum	SCC-Ag	OS, DSS	Elevated serum SCC-Ag independently predicted worse OS and DSS in the oral-cavity subgroup (multivariate HR 1.21).
Tsai [20]	2023	Taiwan ^a^	Prognostic (outcome-associated)	288	Retrospective cohort	Serum	SCC-Ag	DFS, OS	Serum SCC-Ag, isolated from a composite nomogram, independently predicted OS (HR 1.91) and DFS (HR 1.55).
Hsu [13]	2015	Taiwan ^a^	Prognostic (outcome-associated)	130	Retrospective cohort	Serum	CYFRA 21-1, CRP	DFS, OS, distant metastasis	CYFRA 21-1 alone was not independently prognostic; combined with CRP it significantly predicted DFS.
De Paz [21]	2019	Taiwan ^a^	Prognostic (outcome-associated)	246	Retrospective cohort	Serum	SCC-Ag, CRP, CYFRA 21-1	DFS, OS	SCC-Ag and CRP each independently predicted DFS; CYFRA 21-1 alone did not reach significance for DFS or OS.
Malhotra [22]	2016	India	Prognostic (outcome-associated)	50	Cross-sectional	Salivary + serum	CYFRA 21-1	Recurrence, grade	Salivary CYFRA 21-1 was higher in Grade II vs. I tumors and significantly elevated in patients with recurrence.
Zhong [23]	2007	China	Prognostic (outcome-associated)	30	Prospective cohort	Salivary	CYFRA 21-1	Recurrence, OS	Salivary CYFRA 21-1 was elevated vs. controls and significantly higher in patients who later recurred.
Huang [4]	2012	Taiwan ^a^	Prognostic (outcome-associated)	142	Retrospective cohort	Serum	SCC-Ag, CRP	DFS, OS	Combined preoperative SCC-Ag and CRP independently predicted both DFS and OS.
Adel [24]	2016	Taiwan ^a^	Prognostic (outcome-associated)	277	Retrospective cohort	Serum	SCC-Ag, CRP	DFS, OS	Preoperative SCC-Ag and CRP independently predicted DFS and OS.
Chen [25]	2014	Taiwan ^a^	Prognostic (outcome-associated)	100 recurrent/534 cohort	Retrospective cohort	Serum	SCC-Ag, CRP	OS after recurrence	Among patients with recurrent OSCC, elevated SCC-Ag and CRP predicted markedly worse subsequent survival.
Chen [26]	2011	Taiwan ^a^	Prognostic (outcome-associated)	59	Retrospective cohort	Serum	CRP	DFS, OS	Preoperative CRP alone independently predicted DFS but not OS.
Lin [27]	2011	Taiwan ^a^	Prognostic (outcome-associated)	79	Retrospective cohort	Serum	SCC-Ag	DFS, OS	Elevated preoperative SCC-Ag was strongly associated with worse OS.
Stamatoski [28]	2026	North Macedonia	Prognostic (outcome-associated)	40	Prospective cohort	Salivary + serum	CYFRA 21-1	Recurrence-free survival	Both the baseline salivary CYFRA 21-1 level and its post-surgical drop remained significant recurrence predictors after adjustment, while TNM stage and nodal status did not.
Kruse [32]	2010	Switzerland	Prognostic (outcome-associated)	278	Retrospective cohort	Serum	CRP	Recurrence, metastasis	Higher recurrence rate with elevated CRP (RR 1.77), but survival analysis was non-significant; no association with metastasis.
Tai [33]	2017	Taiwan ^a^	Prognostic (outcome-associated)	343	Retrospective cohort	Serum	CRP	OS, DFS	Elevated CRP was significantly associated with OS (HR 2.24) and DFS (HR 1.90) by univariate analysis, strongest in buccal-subsite cancers; no covariate-adjusted model was reported.
Graupp [36]	2018	Austria	Prognostic (outcome-associated)	197	Cohort	Serum	CRP	OS, DFS	CRP independently predicted OS (HR 1.63); DFS significant only in highest quartile.
Yamagata [37]	2020	Japan	Prognostic (outcome-associated)	88	Cohort	Serum	CRP (secondary covariate)	OS	Significant univariately, non-significant after adjustment (HR 0.97, NS).
Ge [44]	2018	China	Prognostic (outcome-associated)	236	Cohort	Serum	CRP (secondary covariate)	OS	Significant univariately, non-significant after adjustment (HR 1.47, NS).
Park [38]	2017	Korea	Prognostic (outcome-associated)	40	Retrospective cohort	Serum	CRP (secondary covariate)	OS	Not independent after adjustment (HR 2.53, NS); COP-MPV score was the primary predictor.
Khandavilli [39]	2009	United Kingdom	Prognostic (outcome-associated)	60	Retrospective cohort	Serum	CRP	OS	Raised CRP independently predicted worse OS (death OR 5.64).
Ito [41]	2023	Japan	Prognostic (outcome-associated)	61 (age ≥ 65 y)	Retrospective cohort	Serum	CRP (secondary covariate)	OS, DFS	Not independent after adjustment (both NS); GNRI was the primary predictor.
Fang [43]	2013	Taiwan ^a^	Prognostic (outcome-associated)	226	Retrospective cohort	Serum	CRP	DFS, OS	Independently predicted both DFS (HR 2.45) and OS (HR 2.54).
Singhavi [42]	2021	India	Cross-sectional/clinicopathological (no outcome association)	69	Prospective cohort	Serum	CRP	Bone erosion, ECS, nodal status, site, stage (narrative)	Correlated with local tumor burden but not with OS.
Metgud [40]	2016	India	Cross-sectional/clinicopathological (no outcome association)	60 (20 OSCC + 20 premalignant + 20 control)	Cross-sectional	Serum + Salivary	CRP	Clinical stage within OSCC (narrative)	CRP rose stepwise from controls to premalignant to OSCC, and with stage.
Acharya [34]	2018	India	Cross-sectional/clinicopathological (no outcome association)	60 (+30 control)	Cross-sectional (case–control)	Serum	CRP	Nodal status (narrative)	CRP correlated with positive nodal status.
Blatt [35]	2018	Germany	Cross-sectional/clinicopathological (no outcome association)	82 (+63 control)	Prospective cohort	Serum	CRP	Stage, nodal status, ECS (narrative)	CRP correlated with stage/nodal status/ECS but not OS.
Krimmel [29]	1998	Germany	Cross-sectional/clinicopathological (no outcome association)	121	Retrospective cohort	Serum	SCC-Ag, CEA, CA19-9, CA125	Relapse (qualitative)	SCC-Ag rose 1–2 months before clinically detected relapse in about half of recurrent cases. CA19-9/CA125 were uninformative; CEA tracked smoking/alcohol habits more than disease status.
Nagumo [30]	1990	Japan	Cross-sectional/clinicopathological (no outcome association)	54	Retrospective	Serum	SCC-Ag	Clinical stage, histologic grade, metastasis	SCC-Ag positivity (42.6%) correlated with T-stage; recurrence was numerically lower in the elevated group (13.3% vs. 16.1%), an inverse pattern. Population confirmed oral-cavity only.
Jablonska [31]	1997	Poland	Cross-sectional/clinicopathological (no outcome association)	42	Cross-sectional	Serum	CRP	Stage correlation (narrative)	Serum CRP correlated with advanced tumor stage.

Abbreviations: OS, overall survival; DFS, disease-free survival; DSS, disease-specific survival; SCC-Ag, squamous cell carcinoma antigen; CRP, C-reactive protein; RR, risk ratio; ECS, extracapsular spread; COP-MPV, count of platelet and mean platelet volume score; OR, odds ratio; GNRI, Geriatric Nutritional Risk Index; NS, not significant. ^a^ Studies sharing the Chang Gung Memorial Hospital registry with overlapping enrolment windows; treated as non-independent and not pooled together.

## Data Availability

The data supporting the findings of this review are available within the article and its Supplementary Material. Any additional data are available from the corresponding author upon reasonable request.

## Supplementary Materials

The following supporting information can be downloaded at: https://www.mdpi.com/article/10.3390/medsci14060610/s1, Figure S1: Not-pooled evidence stratified by sample size, Figure S2: Not-pooled evidence stratified by QUIPS composite study quality, Figure S3: Not-pooled evidence stratified by marker combination, Figure S4: Not-pooled evidence stratified by enrolment era; Table S1: Search strategy, Table S2: Full-text exclusion list with reasons [9,50,52,53,54,55,56,57,58,59,60,61,62,63,64], Table S3: Assessment of the outcomes by GRADE framework [17,18,64], Table S4: Adjustment covariates un multivariable models [4,13,19,20,21,22,23,24,25,26,27,28,29,30,31,32,33,34,35,36,37,38,39,40,41,42,43,44]. File S1: PRISMA 2020 Checklist [14].
