# Supplementary material for: Prognostic Value and Stage/Nodal Correlates of Serum and Salivary CRP, CYFRA 21-1, and SCC-Ag Biomarkers in Oral Squamous Cell Carcinoma: A Systematic Review and Meta-Analysis
Source: Med Sci (Basel). 2026 Sep 27;14(6):610. doi: 10.3390/medsci14060610 (PMC13707081; doi:10.3390/medsci14060610)
Supplement: Supplementary file 1 [file medsci-14-00610-s001.zip › medsci-4539136-supplementary-pub.pdf]

Supplementary material for:

Prognostic value and Stage/Nodal Correlates of Serum and Salivary CRP, CYFRA 21-1, and SCC-Ag biomarkers in Oral Squamous Cell Carcinoma: A Systematic Review and Me-ta-Analysis

Supplementary Table S1. Search strategy

Database	Search String	Results (n)
PubMed (MEDLINE)	("Mouth Neoplasms"[MeSH] OR "Tongue Neoplasms"[MeSH] OR "oral squamous cell carcinoma"[tiab] OR "OSCC"[tiab] OR "oral cancer"[tiab] OR "oral cavity cancer"[tiab] OR "mouth neoplasm*"[tiab] OR "tongue neoplasm*"[tiab] OR "tongue cancer"[tiab])<br>AND<br>("CYFRA 21-1"[tiab] OR "CYFRA21-1"[tiab] OR "Cyfra 21.1"[tiab] OR "cytokeratin 19 fragment"[tiab] OR "CK19 fragment"[tiab] OR "squamous cell carcinoma antigen"[tiab] OR "SCC-Ag"[tiab] OR "SCCA"[tiab] OR "C-reactive protein"[tiab] OR "CRP"[tiab])	244
Embase	('mouth cancer'/exp OR 'tongue cancer'/exp OR 'oral squamous cell carcinoma':ti,ab OR 'OSCC':ti,ab OR 'oral cancer':ti,ab OR 'oral cavity cancer':ti,ab OR 'mouth neoplasm*':ti,ab OR 'tongue neoplasm*':ti,ab)<br>AND<br>('cytokeratin fragment antigen 21'/exp OR 'CYFRA 21-1':ti,ab OR 'CYFRA21-1':ti,ab OR 'Cyfra 21.1':ti,ab OR 'cytokeratin 19 fragment':ti,ab OR 'CK19 fragment':ti,ab OR 'squamous cell carcinoma antigen'/exp OR 'SCC-Ag':ti,ab OR 'C reactive protein'/exp OR 'CRP':ti,ab)<br>AND [humans]/lim	447
Web of Science	(("oral squamous cell carcinoma" OR "OSCC" OR "oral cancer" OR "oral cavity cancer" OR "mouth neoplasm*" OR "tongue neoplasm*" OR "tongue cancer") AND ("CYFRA 21-1" OR "CYFRA21-1" OR "Cyfra 21.1" OR "cytokeratin 19 fragment" OR "CK19 fragment" OR "squamous cell carcinoma antigen" OR "SCC-Ag" OR "C-reactive protein" OR "CRP"))	399
**Total**		**1,090**

Supplementary Table S2. Full-text exclusion list

#	Study	Domain failed	Reason for exclusion
1	Rudhart et al. 2022 [52]	Population	Cohort is oropharyngeal SCC (base of tongue, tonsil, pharyngeal wall), not oral cavity.
2	Fontana et al. 1993 [53]	Exposure + Population	No CYFRA 21-1 measured; mixed oral cavity/oropharynx/hypopharynx cohort with no oral-cavity-only outcome subgroup.
3	Czerninski et al. [54]	Exposure + Study design	No CYFRA 21-1 measured; cross-sectional comparison with no recurrence/OS/DFS follow-up data.
4	Kurokawa et al. 2002 [55]	Exposure	Primary exposure is serum TNF-α; SCC-Ag measured only as a secondary correlate, no independent outcome data.
5	Fischbach et al. 1990 [56]	Population	Mixed "oral and facial" SCC cohort (oropharynx, nose, maxillary sinus alongside oral cavity), no site-stratified outcome data.
6	Karmelic et al. 2022 [57]	Outcome	Pure cross-sectional diagnostic case-control comparison; all patients same T1N0M0 stage by design, so no stage-correlation or survival/recurrence finding possible.
7	Jantharapattana et al. 2018 [58]	Population + Outcome	Only 11/52 (21%) patients are oral cavity; outcome is tumor-volume correlation, not stage/grade/recurrence/survival.
8	Chen et al. 2007 [59]	Exposure	Primary exposure is serum HER-2/neu; SCCA is secondary with only χ^2 statistics, no HR/OR/CI.
9	Yen 1998 [60]	Outcome	Diagnostic case-control accuracy study only; no recurrence, survival, or stage/grade correlation reported.
10	Hedstrom 1999 [61]	Population	Survival analysis pools 53 oral cavity + 6 oropharynx patients with no site-stratified breakout.
11	Gupta et al. 2016 [62]	Outcome	Purely diagnostic cross-sectional group comparison (no lesion vs. potentially malignant disorder vs. OSCC); no correlation of CRP to stage, grade, metastasis, recurrence, or survival within the OSCC group itself.
12	Charakorn et al. 2018 [50]	Population + Exposure	Systematic review/meta-analysis of SCC-Ag in cervical squamous cell carcinoma, not oral cavity OSCC; CRP is not analyzed anywhere in the paper.
13	Rani et al. 2024 [63]	Outcome	Purely diagnostic cross-sectional comparison across 4 groups (leukoplakia, OSMF, oral cancer, healthy control), no within-cancer-group stage/grade/survival correlation.
14	Travassos et al. 2018 [9]	Population + Publication type	Systematic review/meta-analysis of SCC-Ag in mixed head and neck squamous cell carcinoma (7 HNSCC, 2 larynx, 2 oral-only of 11 pooled studies), not a primary study and not an isolable oral-cavity-only population.
15	Yoshimura et al. 1988 [64]	Population	Extra-oral maxillary sinus SCC

SCC-Ag = squamous cell carcinoma antigen, CYFRA 21-1 = cytokeratin 19 fragment, HR = hazard ratio, OR = odds ratio, CI = confidence interval, OS = overall survival, DFS = disease-free survival

Supplementary Table S3. Assessment of the outcomes by GRADE framework

Outcome (marker)	Nº studies	Study design	Risk of bias	Inconsistency	Indirectness	Imprecision	Certainty
Overall survival (serum SCC-Ag ± CRP)	14 (2 pooled SCC-Ag + 5 pooled CRP-alone + 7 non-independent)	Observational cohorts (mixed independent / same-registry)	Serious[a]	Serious[b]	Serious[c]	Serious[d]	⨁○○○ Very low
Disease-free survival (serum CYFRA 21-1 ± SCC-Ag/CRP)	7 unique (2 pooled CYFRA 21-1 + 6 non-independent estimates from 5 further studies; De Paz 2019 contributes to both)	Observational cohorts (mixed independent / same-registry)	Serious[a]	Serious[e]	Serious[c]	Serious[d]	⨁○○○ Very low
Recurrence (salivary CYFRA 21-1; serum CRP)	4 (2 pooled SMD + 1 standalone HR [salivary CYFRA] + 1 standalone RR pair [serum CRP])	Observational cohorts (mixed independent)	Serious[f]	Serious[g]	Serious[h]	Serious[i]	⨁○○○ Very low

Each row combines independent-cohort pooled estimate(s) with non-independent (same-registry) or standalone evidence for that outcome into a single certainty judgement, using both formal GRADE [17, 64] and modified GRADE for evidence without a single pooled estimate [18].

a Risk of bias: Pooled studies were mostly low-to-moderate risk on QUIPS, though four of five CRP-alone OS studies treated CRP as a secondary co-variable rather than the primary exposure. All rows also included non-independent same-registry studies rated Moderate or worse on confounding. One level lower.

b Inconsistency (OS): Both pooled single-marker estimates were non-significant and heterogeneous (SCC-Ag: HR 1.43, 95% CI 0.09–23.14, I^2=66%, k=2; CRP: HR 1.33, 95% CI 0.89–1.99, I^2=52%, k=5), while the seven non-independent studies agreed in direction (HR>1 in 7/7, 6 significant) but varied widely in magnitude (HR 1.82–11.11). One level lower.

c Indirectness: Each row mixes non-identical exposures. For OS: separately pooled SCC-Ag-alone and CRP-alone estimates plus a composite SCC-Ag+CRP panel in the non-independent evidence, with four of five CRP-alone studies measuring CRP only as a secondary co-variable. For DFS: a pooled CYFRA 21-1-alone estimate versus composite 2–3-marker panels, plus a CRP-alone secondary-co-variable estimate in the non-independent evidence. All rows dropped one level.

d Imprecision: All pooled CIs crossed the null (SCC-Ag OS: 0.93–2.18; CRP OS: 0.94–1.89; CYFRA DFS: 0.39–6.61); non-independent/standalone evidence adds direction but not precision, and for DFS now includes a wide-CI non-significant estimate (Ito 2023, HR 1.38, 95% CI 0.57–3.35). One level lower.

e Inconsistency (DFS): The pooled CYFRA 21-1 estimate (HR 1.61, borderline, I^2=0%) and most composite-marker studies (HR 2.41–4.05) agree in direction, but a non-significant single-marker estimate (Ito 2023, HR 1.38, 95% CI 0.57–3.35) narrows this agreement, and composite-marker effects remain consistently larger than single-marker ones. One level lower.

f Risk of bias (recurrence): The two SMD-contributing salivary CYFRA 21-1 studies compare unadjusted group averages; the standalone salivary CYFRA study is well-conducted but cannot offset this. A standalone serum CRP study reports a crude, unadjusted recurrence risk ratio that conflicts with its own adjusted, non-significant Kaplan-Meier/log-rank analysis for the same comparison. One level lower.

g Inconsistency (recurrence): Salivary CYFRA 21-1 evidence consistently suggests a positive recurrence association, but serum CRP shows directionally opposite findings across its two outcomes (recurrence RR 1.77 vs. metastasis RR 0.55, NS) and across three non-comparable effect measures (SMD, HR, RR). One level lower.

h Indirectness (recurrence): Combining an SMD, HR, and RR in one row is substantially indirect, further compounded by spanning two mechanistically distinct markers (salivary CYFRA 21-1; serum CRP). One level lower.

i Imprecision (recurrence): The pooled SMD sample (n=74) and the independent salivary CYFRA study (n=40) are too small for precision; the CRP evidence relies on a crude 2×2 event count (21/85 vs. 27/193) with a wide CI. One level lower.

Supplementary Table S4. Adjustment covariates un multivariable models

Study	Outcome / panel	Adjustment covariates (or adjustment status)
Imai et al [19]	OS – SCC-Ag (pooled)	T classification, N classification, M classification, age
Tsai et al [20]	OS/DFS – SCC-Ag (pooled)	AJCC stage, tumor differentiation, Charlson Comorbidity Index (CCI); SCC-Ag modeled separately from the composite inflammatory index to avoid multicollinearity
Hsu et al [13]	DFS/OS – CYFRA 21-1, CRP (non-pooled, registry)	Not reported — the paper states a significant adjusted (combined CYFRA 21-1 + CRP) result but does not tabulate the covariates included in the model
De Paz et al [21]	DFS/OS – SCC-Ag, CRP, CYFRA 21-1 (non-pooled, registry)	Pathologic T status, nodal status with extracapsular spread, tumor differentiation, tumor depth
Huang et al [4]	DFS/OS – SCC-Ag, CRP (non-pooled, registry)	Tumor differentiation, tumor depth, nodal status, tumor status
Adel et al [24]	DFS/OS – SCC-Ag, CRP (non-pooled, registry)	Age, differentiation, tumor depth, tumor status, nodal status, lymph node density
Chen et al (2014) [25]	OS after recurrence – SCC-Ag, CRP (non-pooled, registry)	Age, overall recurrent tumor stage (a composite stage variable was used instead of separate T/N/M terms, specifically to limit multicollinearity)
Chen et al (2011) [26]	DFS/OS – CRP alone (non-pooled, registry)	Tumor status (T1/T2 vs T3/T4), lymph node metastasis, lymph node extracapsular spread (LNECS) — significant for DFS (adjusted HR 3.124, 95% CI 1.041–9.376, p=0.042), not significant for OS (adjusted HR 1.819, 95% CI 0.500–6.612, p=0.364)
Lin et al [27]	DFS/OS – SCC-Ag (non-pooled, registry)	Tumor stage, nodal metastasis
Malhotra et al [22]	Recurrence – CYFRA 21-1 (pooled, SMD)	No adjustment — group comparisons only (Cyfra 21-1 levels by histologic grade I vs II, and by recurrence vs no recurrence); no Cox/regression-adjusted model reported
Zhong et al [23]	Recurrence – CYFRA 21-1 (pooled, SMD)	No adjustment — univariate group-mean comparison only, no multivariable model
Stamatoski et al [28]	Recurrence-free survival – CYFRA 21-1 (non-pooled, k=1)	AJCC stage, nodal status, risk factors, treatment type
Kruse et al [32]	Recurrence, metastasis – CRP (non-pooled)	No adjustment — crude recurrence rate ratio from raw proportions, not a regression model

Tai et al [33]	OS/DFS – CRP (non-pooled, registry)	No adjustment — the paper's own Methods state only chi-square testing and log-rank survival comparison; the reported HRs (DFS 1.902 [1.302–2.778]; OS 2.235 [1.393–3.585], overall and by subsite) are explicitly presented under 'univariate analysis' and no Cox multivariate model is reported anywhere in the paper
Graupp et al [36]	OS/DFS – CRP (pooled, CRP-alone OS)	T stage (T1+T2 vs T3+T4), N stage (N0 vs N+) — entered together with CRP in the multivariate Cox model; N/L-ratio was tested but not significant on univariate analysis and was excluded from the multivariate model
Yamagata et al [37]	OS – CRP (pooled, CRP-alone OS)	D-dimer, age, neutrocyte count, neutrophil-lymphocyte ratio (NLR), management (surgery vs radiotherapy) — all entered together with CRP in the multivariate Cox model; management was the only factor that remained independently significant
Ge et al [44]	OS – CRP (pooled, CRP-alone OS)	Tumor size, nodal metastasis, RDW, hemoglobin, albumin, SCC-Ag, adjuvant therapy — all entered together with CRP in the multivariate Cox model; RDW was the only factor that remained independently significant
Park et al [38]	OS – CRP (pooled, CRP-alone OS)	N-metastasis, COP-MPV score, platelet count, platelet-lymphocyte ratio (PLR) — all entered together with CRP in the multivariate Cox model; N-metastasis and COP-MPV score were the only factors that remained independently significant
Khandavilli et al [39]	OS – CRP (non-pooled, k=1)	No adjustment — odds ratio calculated from raw death counts, not a regression model
Ito et al [41]	OS/DFS – CRP (non-pooled, k=1)	Alcohol use, GNRI, tumor stage, tumor differentiation (grade), lymphovascular invasion, vascular invasion, perineural invasion, close margin status, postoperative treatment — all entered together with CRP in the multivariate Cox model; GNRI (and, for DFS, postoperative treatment) were the only factors that remained independently significant
Fang et al [43]	DFS/OS – CRP (non-pooled, registry)	Tumor differentiation, tumor depth, nodal status, pathologic tumor status (combined CRP × NLR stratification model)
Singhavi et al [42]	Bone erosion, ECS, nodal status, site, stage (narrative)	Not applicable — cross-sectional correlation, no regression-adjusted model
Metgud et al [40]	Clinical stage (narrative)	Not applicable — cross-sectional group comparison, no regression-adjusted model
Acharya et al [34]	Nodal status (narrative)	Not applicable — cross-sectional correlation, no regression-adjusted model
Blatt et al [35]	Stage, nodal status, ECS (narrative)	Not applicable — cross-sectional/cohort correlation, no regression-adjusted model
Krimmel et al [29]	Relapse timing (narrative, qualitative)	Not applicable — descriptive/qualitative correlation, no regression-adjusted model
Nagumo et al [30]	Clinical stage, histologic grade, metastasis (narrative)	Not applicable — cross-sectional correlation, no regression-adjusted model
Jablonska et al [31]	Stage correlation (narrative)	Not applicable — cross-sectional correlation, no regression-adjusted model

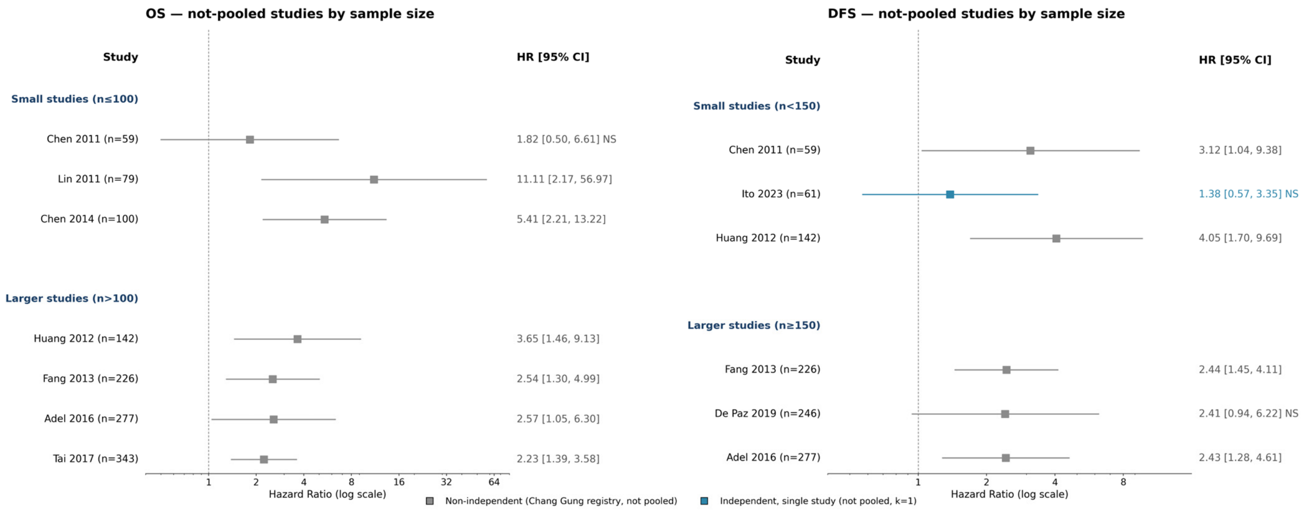

Supplementary Figure S1. Not-pooled evidence stratified by sample size

Individual point estimates from the non-independent, not-pooled studies grouped descriptively into smaller and larger sample studies for side-by-side comparison.

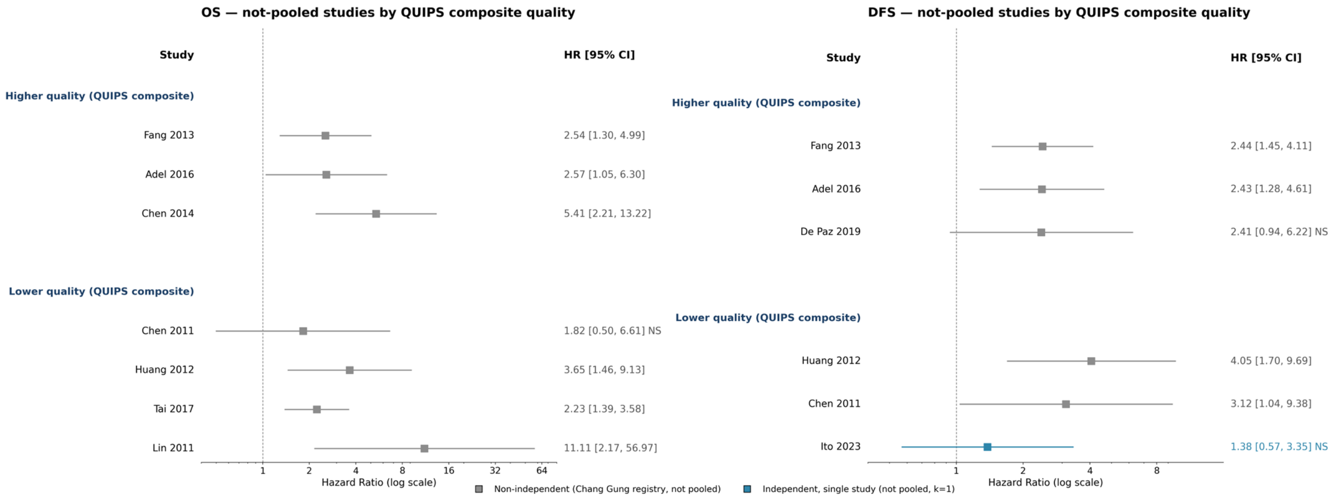

Supplementary Figure S2. Not-pooled evidence stratified by QUIPS composite study quality

Individual point estimates from the non-independent, not-pooled studies grouped descriptively by study quality evaluated by QUIPS.

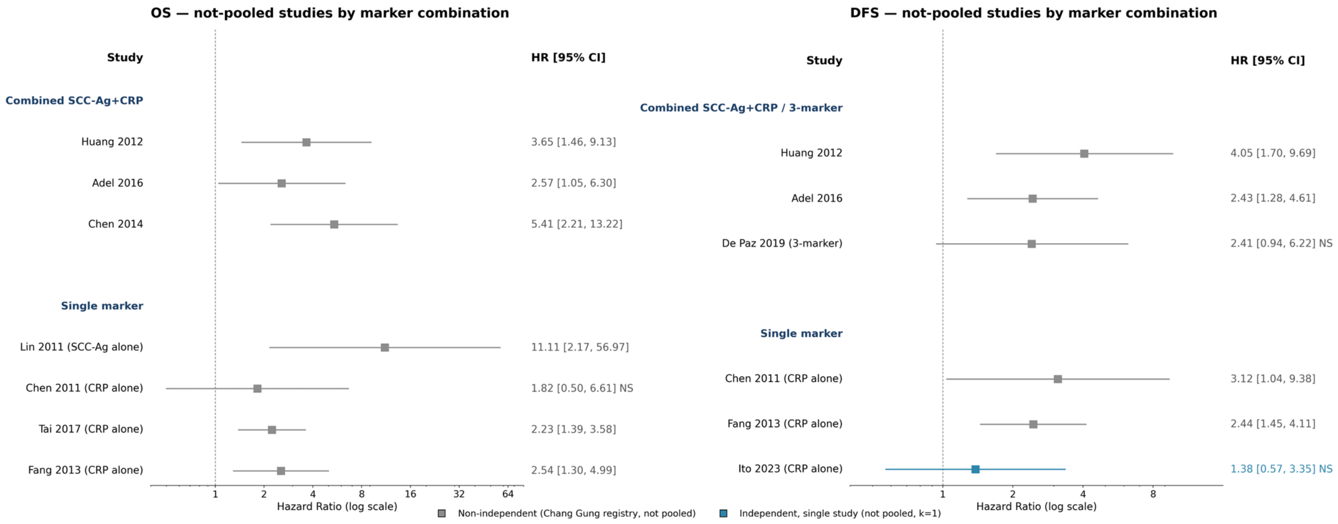

Supplementary Figure S3. Not-pooled evidence stratified by marker combination

Individual point estimates from the non-independent, not-pooled studies grouped descriptively by single marker compared to combined marker model.

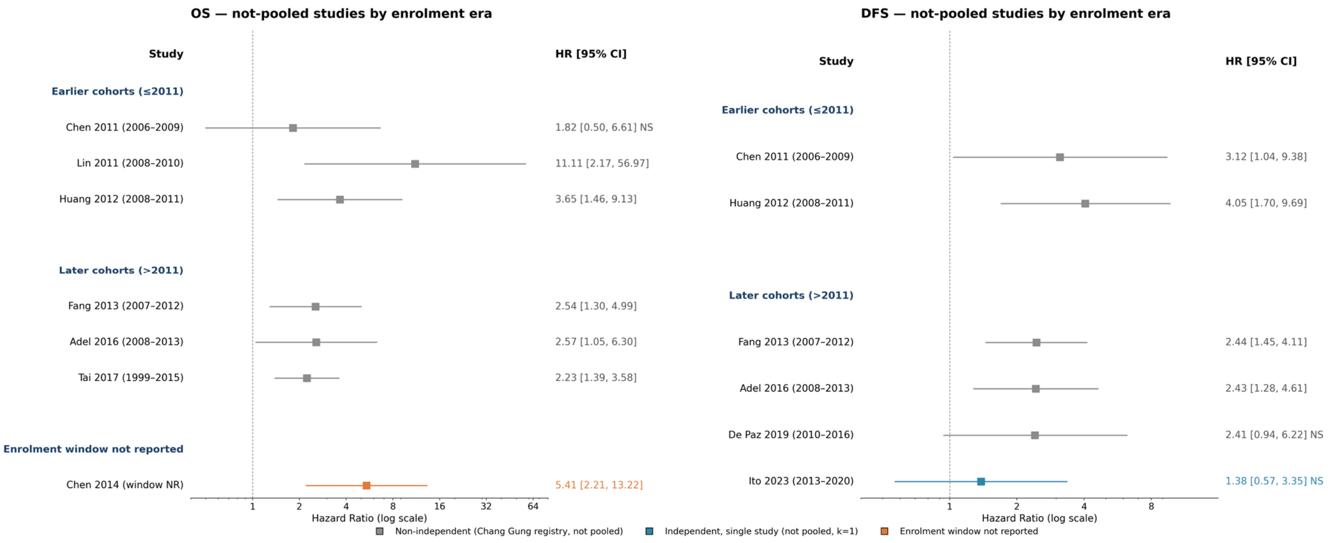

Supplementary Figure S4. Not-pooled evidence stratified by enrolment era

Individual point estimates from the non-independent, not-pooled studies grouped descriptively by enrolment closure year (≤2011 vs >2011).
